# Identification of Immune-Related Subtypes and Construction of a Novel Prognostic Model for Bladder Urothelial Cancer

**DOI:** 10.3390/biom12111670

**Published:** 2022-11-11

**Authors:** Jiange Zhang, Caisheng Huang, Rirong Yang, Xiang Wang, Bo Fang, Junhao Mi, Hao Yuan, Zengnan Mo, Yihai Sun

**Affiliations:** 1 Department of Urology, The First Affiliated Hospital of Guangxi Medical University, Nanning 530021, China; 2 Center for Genomic and Personalized Medicine, Guangxi Medical University, Nanning 530021, China; 3 Guangxi Collaborative Innovation Center for Genomic and Personalized Medicine, Nanning 530021, China; 4 Guangxi Key Laboratory for Genomic and Personalized Medicine, Guangxi Key Laboratory of Colleges and Universities, Nanning 530021, China; 5 Department of Urology, The Second Affiliated Hospital of Guangxi Medical University, Nanning 530007, China; 6 Department of Urology, The Nanning Second People’s Hospital, The Third Affiliated Hospital of Guangxi Medical University, Nanning 530031, China; 7 Department of Immunology, School of Basic Medical Sciences, Guangxi Medical University, Nanning 530021, China; 8 Collaborative Innovation Center of Regenerative Medicine and Medical BioResource Development and Application Co-Constructed by the Province and Ministry, Guangxi Medical University, Nanning 530021, China

**Keywords:** bladder urothelial cancer (BLCA), prognosis, immunity, survival, immune-related prognosis model (IRPM)

## Abstract

The purpose of this study was to explore the relationship between bladder urothelial cancer (BLCA) and immunity, to screen prognosis-related immune genes (PIGs), and to construct an immune-related prognosis model (IRPM). We processed the relevant data of The Cancer Genome Atlas (TCGA-BLCA) and GSE13507 using R software and Perl. We divided BLCA into high-immunity and low-immunity subtypes. There were significant differences in the two subtypes. In addition, we identified 13 PIGs of BLCA by jointly analyzing the gene expression data and survival information of GSE13507 and TCGA-BLCA, and constructed IRPM through nine of them. The low-risk group had better survival outcome than the high-risk group. We also constructed a nomogram based on clinicopathological information and risk scores of the patients. Moreover, the prognosis of BLCA patients was significantly impacted by the expression of almost every gene used to calculate the risk score. The result of real-time fluorescence quantitative polymerase chain reaction revealed that all the genes used to calculate the risk score were differentially expressed between BLCA and adjacent normal tissues, except PDGFRA. Our research provided potential targets for the treatment of BLCA and a reference for judging the prognosis of BLCA.

## 1. Introduction

Bladder cancer is the second most common tumor in the urinary system [1], and bladder urothelial cancer (BLCA) is the most common pathological type of bladder cancer, accounting for about 90% of bladder cancer [2]. The tumor microenvironment (TME) of BLCA is obviously heterogeneous [3,4], and the prognosis of advanced BLCA is very poor, although good progress has been made in targeted therapy and neoadjuvant chemotherapy of BLCA [5]. There are two main types of BLCA: non-muscle invasive bladder cancer (NMIBC) and muscle invasive bladder cancer (MIBC). Most patients with BLCA can be diagnosed at the stage of NMIBC, but the rate of recurrence and progression is still high, with about 78% of patients relapsed within 5 years [6]. This is due to the lack of effective biomarkers or tools to accurately judge the clinical response and prognosis of patients with BLCA. Therefore, the development and application of various biological detection and informatics techniques to show the difference in prognosis of BLCA patients according to their biological heterogeneity is essential for the treatment of BLCA and improvement of its prognosis [7,8,9].

TME is a heterogeneous system composed of cancer cells, extracellular matrix and immune cells, and other molecules [3,8,10]. Even in patients with the same pathological stage and grade, patients have different clinical responses to the same treatment, which may be closely related to the high heterogeneity of TME [11,12]. The high heterogeneity of TME in BLCA hinders the progress of accurate prediction of BLCA prognosis and accurate treatment of BLCA [8]. Therefore, accurate identification of TME heterogeneity can further accurately judge the prognosis of BLCA patients and promote the treatment development of BLCA. Immunity is an important part of TME. Understanding the immune-related characteristics of BLCA is of great significance for its risk stratification and targeted therapy [13,14]. Up to now, although many studies have analyzed BLCA patients from the perspective of immune-cell infiltration [15,16,17], there is a lack of joint exploration of the relationship between BLCA and immunity from many immune aspects, such as immune-related genes, immune-cell infiltration, and transcription factors (TFs).

In this study, tumor samples from The Cancer Genome Atlas (TCGA-BLCA) were split into high-immunity (Immunity-H) and low-immunity (Immunity-L) subtypes through single-sample gene set enrichment analysis. Moreover, we systematically analyzed the relationships between tumor and immune cells, immune genes, and HLA genes. In addition, we scored the tumor samples of the Gene Expression Omnibus (GEO, GSE13507) and TCGA-BLCA by the expression levels of nine prognosis-related immune genes (PIGs) and divided the samples into a high-risk group and low-risk group according to the median risk score. The immune-related prognosis model (IRPM) was constructed through the tumor samples of TCGA-BLCA and GSE13507. What is more, we designed a nomogram to facilitate clinicians to quickly assess the prognosis of BLCA patients. Finally, we investigated the influence of nine genes related to the risk score on the survival time of BLCA patients through the Kaplan–Meier Plotter database and detected the differences in the expression of these genes between BLCA tissues and normal tissue adjacent to cancer using real-time fluorescence quantitative polymerase chain reaction (RT-qPCR).

## 2. Materials and Methods

### 2.1. Download and Processing of Data

The Gene Expression Omnibus (GEO, https://www.ncbi.nlm.nih.gov/geo/, accessed on 6 March 2022)) and The Cancer Genome Atlas (TCGA, https://portal.gdc.cancer.gov, accessed on 6 March 2022)) are public databases that contain gene expression data for a variety of cancers, and the TCGA also contains detailed clinical information of patients and data such as tumor mutation burden (TMB), providing a wide range of resources for scientists around the world to study tumors. Transcriptome data (HTSeq-FPKM, n = 411), clinical information (n = 409), and TMB information (n = 405) of BLCA were downloaded from TCGA database. The disease types we chose to download were transitional cell papillomas and carcinomas. In addition, transcriptome data and prognostic parameters of BLCA samples were downloaded through the GEO (GSE13507, n = 165). We processed the related data of TCGA-BLCA and GSE13507 through R (4.0.4) and Perl (5.30.0.1) software, making the data easy to understand and visualize.

### 2.2. Hierarchical Clustering of TCGA-BLCA Samples

Single-Sample Gene Set Enrichment Analysis (ssGSEA) was performed on TCGA-BLCA samples based on signature genes representing 29 immune-related functions or immune cells using R packages (“GSVA”, “limma”, “GSEABase”) [18]. Those signature genes are shown in Supplementary File S1. According to the immune characteristics of 411 TCGA-BLCA samples, the samples were divided into two subtypes, including Immunity_H and Immunity_L, using Euclidean distance and Ward’s linkage through R packages (“sparcl”, “Rtsne”, and “ggplot2”) [14].

### 2.3. Further Evaluation of Immune-Related Characteristics and Tumor Purity of the Two Subtypes

The immune and stromal cell infiltrations of 411 TCGA-BLCA samples were assessed using an R package (“estimate”), and the ImmuneScore, TumorPurity, StromalScore, and ESTIMATEScore of the two subtypes were determined [19]. Moreover, the compositions of 22 different kinds of immune cells in the two subtypes were calculated by the CIBERSORT method [20], and the expressions of HLA genes between the two subtypes were compared using R packages (“limma”, “reshape2”, “ggpubr”, “ggplot2”). Meanwhile, we downloaded the list of immune genes from ImmPort (https://www.immport.org, accessed on 12 March 2022)) and intersected these immune genes with the differentially expressed genes (DEGs) of the two subtypes, thus obtaining the differentially expressed immune genes (DEIGs) of the two groups [21,22].

### 2.4. Comparative Analysis of KEGG Pathways of the Two Subtypes

GSEA (Version4.1.0) software was used to analyze the enrichment of the KEGG pathway in the two subtypes. The results of GSEA were transformed into a visualized bubble chart and bar chart using R packages (“ggplot2”, “reshape”). *p*-value < 0.05 was considered statistically significant.

### 2.5. Screening and Analyzing of Prognosis-Related Immune Genes for TCGA-BLCA and GSE13507

We corrected and merged the gene expression data of GSE13507 and TCGA-BLCA. Next, based on the DEIGs of the two subtypes of TCGA, a univariate Cox proportional hazard regression analysis of the merged data of TCGA-BLCA and GSE13507 was carried out with R packages (“survival”, “limma”, “sva”), and the prognosis-related immune genes (PIGs) of BLCA were obtained. The threshold for screening PIGs of BLCA was *p* < 0.001. A hazard radio (HR) > 1 indicated that the gene was a high-risk gene, and HR < 1 indicated that the gene was a low-risk gene. Then, the transcription factors related to cancer progression were identified by CISTROME (http://www.cistrome.org, accessed on 27 March 2022)) [23,24], and the two subtypes of DEGs were used to screen the differentially expressed transcription factors (DETFs). What is more, using R packages (“limma”, “ggalluvial”, “ggplot”, “dplyr”), we constructed the correlation regulation network of PIGs and DETFs through Pearson’s correlation coefficient analysis. False discovery rate (FDR) < 0.001 and |cor| (correlation coefficient) > 0.4 were the thresholds of significant correlation. For the correlation expression of DETFs and PIGs, we also used STRING (https://cn.string-db.org, accessed on 27 March 2022)) for protein–protein interaction (PPI) analysis to reconfirm the associations between them.

### 2.6. Construction of a Prognostic Model for BLCA

Data extracted from the TCGA-BLCA and GSE13507 datasets were used to construct an immune gene-related prognostic model (IRPM) for predicting the prognosis of BLCA. The optimal IRPM of BLCA was created using a LASSO Cox regression model to identify genes linked to survival in the TCGA-BLCA and GSE13507 datasets. We calculated the risk score of each patient based on the correlation coefficient of LASSO Cox regression analysis and the corresponding gene expression of each sample. The risk score was determined using the formula below: the risk score = $\sum_{\gamma }^{n}$ Expression_(γ)_ × coefficient_(γ)_. The gene expression level in the sample was represented by Expression_(γ)_, and the coefficient_(γ)_ represented the regression coefficient of gene γ. According to the median value of the risk score, tumor patients from TCGA-BLCA and GSE13507 were separated into two groups, a high-risk group and low-risk group, and the impact of risk score on the survival of BLCA patients was evaluated using R packages (“survminer”, “survival”). In addition, to reflect the IRPM-based risk characteristic’s forecasting capacity, we developed a receiver operating characteristic curve (ROC) based on time and estimated the area under the curve (AUC) of 1-year, 3-year, and 5-year survival time using R packages (“timeROC”, “rms”, “survminer”, “survival”). Through Spearman correlation analysis, the analysis covered the relationships between TMB and immune-cell infiltration and IRPM-based risk characteristics. Furthermore, multivariate and univariate Cox regression analyses were carried out for sex, age, stage, and risk score. Finally, we constructed a nomogram according to risk score, age, sex, TNM classification, and clinical stage, and performed ROC curve analysis and calibration curve analysis on the proposed nomogram to determine its accuracy.

### 2.7. Kaplan–Meier Plotter and RT-qPCR

Last but not least, the effect of nine genes used to calculate the risk score on the prognosis of BLCA was verified again by the Kaplan–Meier Plotter (https://kmplot.com/analysis, accessed on 30 March 2022)) database. Moreover, we collected five patients’ samples from the Second Affiliated Hospital of Guangxi Medical University to compare the differences in the expression of genes used to calculate the risk score between BLCA and normal tissue adjacent to cancer. The normal bladder tissues of the five samples were obtained at least 2 cm away from the BLCA tissues. Fresh tissue samples were transferred to the laboratory on ice within 20 min and then preserved at −80 °C. The patients’ clinical information is shown in Table 1. We extracted RNA from BLCA and adjacent normal tissues according to the instructions of RNA extraction kit (Vazyme, RC112-01). Then, reverse transcription was performed to synthesize cDNA (Takara, RR037A). Polymerase chain reaction was conducted using SYBR qPCR Master Mix (Vazyme, Q711-03) and LightCycler 96 Instrument (Roche). Each sample was set to repeat detection three times. We calculated the relative expression levels of target genes between BLCA and normal tissue adjacent to cancer through the 2^−ΔΔCt^ method. Rt-qPCR was performed using the following primers.

β-Actin:
5′-TGGACATCCGCAAAGACCTG-3′ (Forward),
5′-CCGATCCACA CGGAGTACTT-3′ (Reverse);
CTSS (Cathepsin S):
5′-TGACAACGGCTTTCCAGTACA-3′ (Forward),
5′-GGCAGCACGATATTTTGAGTCAT-3′ (Reverse);
FABP6 (Fatty Acid Binding Protein 6):
5′-GCCCGCAACTTCAAGATCG-3′ (Forward),
5′-CCTTGCCAACAGTGAACTTGT-3′ (Reverse);
NRP2 (Neuropilin 2):
5′-CCAACGGGACCATCGAATCTC-3′ (Forward),
5′-CCAGCCAATCGTACTTGCAGT-3′ (Reverse);
PDGFRA (Platelet-Derived Growth Factor Receptor, Alpha Polypeptide):
5′-TTGAAGGCAGGCACATTTACA-3′ (Forward),
5′-GCGACAAGGTATAATGGCAGAAT-3′ (Reverse);
PDGFRB (Platelet-Derived Growth Factor Receptor, Beta Polypeptide):
5′-AGCACCTTCGTTCTGACCTG-3′ (Forward),
5′-TATTCTCCCGTGTCTAGCCCA-3′ (Reverse);
S100A7 (S100 Calcium Binding Protein A7):
5′-ACGTGATGACAAGATTGACAAGC-3′ (Forward),
5′-GCGAGGTAATTTGTGCCCTTT-3′ (Reverse);
S100A8 (S100 Calcium Binding Protein n A8):
5′-ATGCCGTCTACAGGGATGAC-3′ (Forward),
5′-ACTGAGGACACTCGGTCTCTA-3′ (Reverse);
S100A9 (S100 Calcium Binding Protein A9):
5′-GGTCATAGAACACATCATGGAGG-3′ (Forward),
5′-GGCCTGGCTTATGGTGGTG-3′ (Reverse);
S100A10 (S100 Calcium Binding Protein A10):
5′-GGCTACTTAACAAAGGAGGACC-3′ (Forward),
5′-GAGGCCCGCAATTAGGGAAA-3′ (Reverse);

## 3. Results

### 3.1. Preliminary Evaluation of the Two Subtypes of BLCA

**Figure 1:**
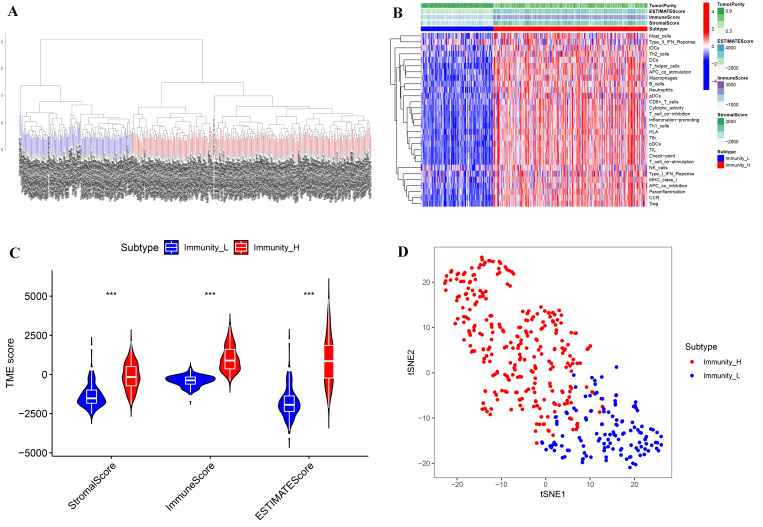
Immune subtypes and clustering in BLCA patients. (**A**) Based on the results of ssGSEA, BLCA patients were divided into Immunity_H and Immunity_L by a hierarchical clustering algorithm. (**B**) Immune infiltration and tumor microenvironment landscape of TCGA-BLCA patients. (**C**) The comparison of ImmuneScore, StromalScore, and ESTIMATEScore in Immunity_H and Immunity_L. (**D**) Verification of immune subtypes by tSNE. *** *p* < 0.001.

A total of 411 tumor samples (transitional cell papillomas and carcinomas) derived from the TCGA-BLCA cohort were divided into two subtypes (Immunity_H and Immunity_L) based on 29 immune gene sets along with the ssGSEA algorithm (Figure 1A,B). R packages (“estimate”, “limma”) were used to calculate the ImmuneScore, StromalScore, ESTIMATEScore, and TumorPurity of the two subtypes. Compared with Immunity_L, the TumorPurity of Immunity_H was lower, however, the ImmuneScore, StromalScore, and ESTIMATEScore of Immunity_H were higher (Figure 1B). Moreover, the violin plot also showed significant differences between the two subtypes in ImmuneScore, ESTIMATEScore, and StromalScore (Figure 1C, wilcox.test). In addition, we further used the tSNE algorithm for clustering analysis of TCGA-BLCA and obtained similar classification results (Figure 1D).

### 3.2. Comparison of Immune-Related Characteristics between Two Subtypes

**Figure 2:**
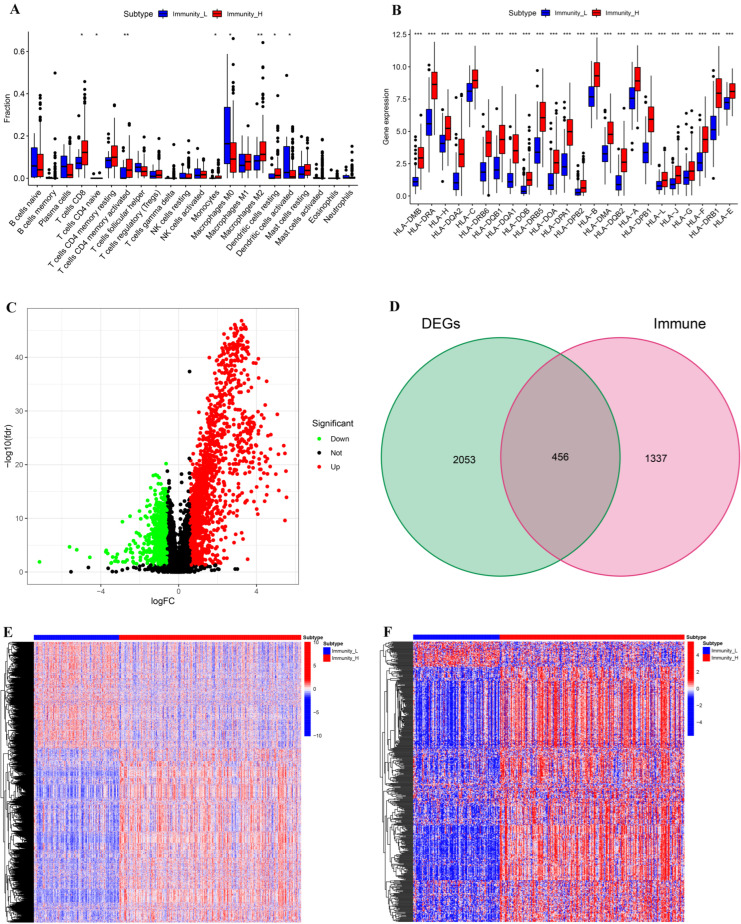
Comparative analyses of immune-cell infiltration, HLA gene expression, DEGs, and DEIGs in the two subtypes. (**A**) Comparison of the immune-cell infiltration between Immunity_H and Immunity_L. (**B**) Comparison of the expression levels of HLA genes between two subtypes. (**C**) Volcano plot of DEGs (Immunity_H vs. Immunity_L); |LogFC| >0.585 and FDR <0.05. (**D**) Venn diagram showing the intersection of DEGs and immune genes. (**E**) Landscape of DEGs between two subtypes. (**F**) Landscape of DEIGs between two subtypes. * *p* < 0.05; ** *p* < 0.01; *** *p* < 0.001.

The fractions of 22 types of immune cells in two subtypes were compared by the CIBERSORT algorithm. Compared with Immunity_L, the fractions of CD8 + T cells, CD4+ naive T cells, CD4+ activated memory T cells, monocytes, M2 macrophages, and resting dendritic cells in Immunity_H were considerably higher, but the proportions of M0 Macrophages and activated Dendritic cells in Immunity_H were considerably lower (Figure 2A). We then investigated the differences in HLA gene expressions between the two subtypes and discovered that Immunity_H had much higher HLA gene expressions than Immunity—L (Figure 2B). These findings illustrated the significance of our classification of BLCA into two subtypes which could largely distinguish the characteristics of BLCA.

Meanwhile, we compared the DEGs of the two subtypes and investigated the DEIGs depending on Immport database. The threshold for screening DEGs between the two subtypes was set to |logFC| > 0.585 and FDR < 0.05. There were more upregulated DEGs in Immunity_H than in Immunity_L (Figure 2C). A total of 2509 genes, including 456 DEIGs, were identified as DEGs of the two subtypes (Figure 2D). The heat maps of DEGs and DEIGs of the two subtypes are shown in Figure 2E,F.

### 3.3. GSEA Enrichment Analysis

**Figure 3:**
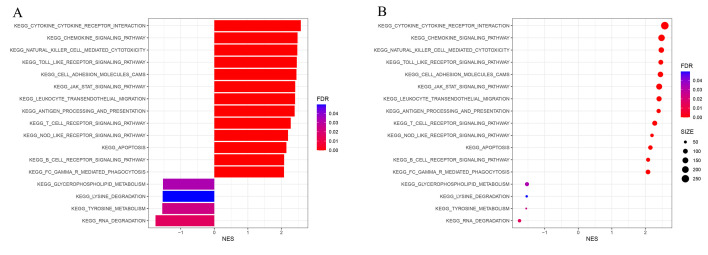
GSEA enrichment analysis. The bar plot (**A**) and dot plot (**B**) show KEGG pathway analysis of DEGs between two subtypes.

The KEGG pathway analysis of GSEA showed that the genes in Immunity_H were mainly related to CYTOKINE RECEPTOR INTERACTION, CELL ADHESION MOLECULE CAMS, NATURAL KILLER CELL-MEDIATED CYTOTOXICITY, TOLL-LIKE RECEPTOR SIGNALING PATHWAY, CHEMOKINE SIGNALING PATHWAY, LEUKOCYTE TRANSENDOTHELIAL MIGRATION, JAK STAT SIGNALING PATHWAY, ANTIGEN PROCESSING AND PRESENTATION, T-CELL RECEPTOR SIGNALING PATHWAY, NOD-LIKE RECEPTOR SIGNALING PATHWAY, APOPTOSIS, B-CELL RECEPTOR SIGNALING PATHWAY, and FC-GAMMA RECEPTOR-MEDIATED PHAGOCYTOSIS, while the genes in Immunity_L were mainly related to GLYCEROPHOSPHOLIPID METABOLISM, LYSINE DEGRADATION, TYROSINE METABOLISM, and RNA DEGRADATION (Figure 3A,B). These findings demonstrate that the KEGG pathway between the two subtypes differed significantly from one another, and further clarified the necessity and feasibility of dividing BLCA into two subtypes.

### 3.4. Identifying PIGs and Constructing Regulatory Networks of PIGs with TFs

**Figure 4:**
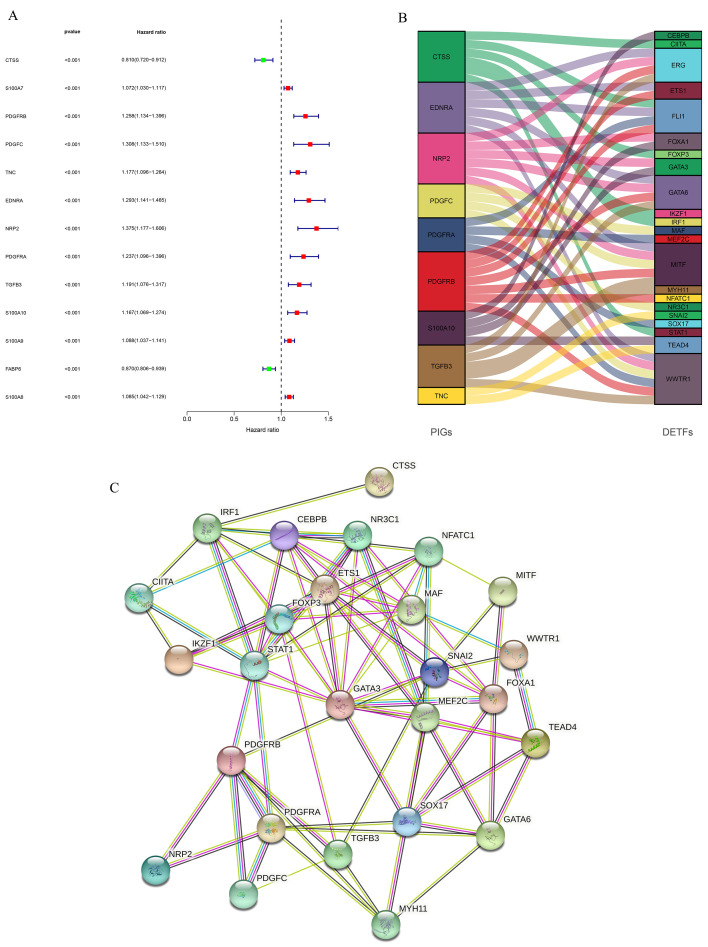
Identification of PIGs and analyses of regulatory networks and interactions between PIGs and DETFs. (**A**) Forest plot based on univariable Cox proportional hazard regression analysis showed the PIGs and their hazard ratios. (**B**) Alluvial diagram of the PIGs and DETFs revealed their regulatory network. (**C**) PPI network between PIGs and DETFs.

We then jointly analyzed the survival data and gene expression data of GSE13507 and TCGA-BLCA, and authenticated PIGs of BLCA through univariate Cox proportional hazard regression analysis. In order to determine the reliability of the identified genes, we set the threshold of the *p*-value as less than 0.001, 13 PIGs were identified, and their hazard ratio (HR) was calculated (Figure 4A). Among the 13 PIGs, there are 2 low-risk genes (HR < 1), namely, CTSS and FABP6, and 11 high-risk genes (HR > 1), namely, S100A7, PDGFRB, PDGFC (Platelet Derived Growth Factor C), TNC (Tenascin C), EDNRA (Endothelin Receptor Type A), NRP2, PDGFRA, TGFB3 (Transforming Growth Factor Beta 3), S100A10, S100A9, and S100A8. Then, we conducted co-expression analysis of these PIGs and DETFs to further understand the initiation and progression of BLCA. NRP2 expression was negatively associated with GATA6 and FOXA1, and S100A10 expression was negatively associated with GATA3 and FOXA1, while other PIGs were positively correlated with the expression of DETFs (Table S1). The Figure 4B showed the regulatory network of PIGs with DETFs. To further confirm the significant correlations between the identified PIGs and DETFs, we performed PPI analysis using STRING and found complex interactions between these DETFs and PIGs (Figure 4C).

### 3.5. Constructing an IRPM of BLCA through Combining Tumor Samples of TCGA-BLCA and GSE13507

**Figure 5:**
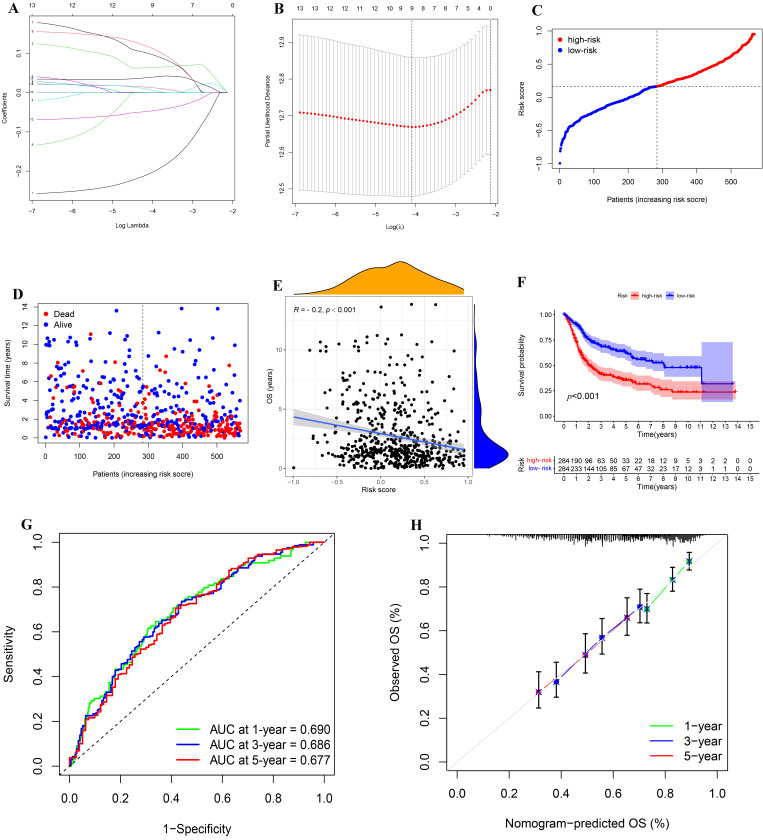
Construction and analysis of IRPM. (**A**) The LASSO coefficient curve drawn when the simulation parameter was set to 1000. (**B**) Cross-validation for tuning parameter selection in the LASSO model. (**C**,**D**) Distribution of risk score, survival time, and survival status in the combined TCGA-BLCA and GSE13507 cohorts. (**E**) Correlation analysis of OS and risk score in the combined TCGA-BLCA and GSE13507 cohorts. (**F**) Survival analysis for different risk groups in the combined TCGA-BLCA and GSE13507 cohorts. (**G**) Prediction of 1-, 3-, and 5-year ROC and AUC based on IRPM in the combined TCGA-BLCA and GSE13507 cohorts. (**H**) The calibration diagram based on IRPM in the combined TCGA-BLCA and GSE13507 cohorts.

We performed LASSO Cox regression analysis on 13 PIGs (Figure 5A,B). Combined with the TCGA-BLCA and GSE13507, we finally obtained an IRPM containing nine PIGs to predict overall survival (OS) of BLCA. Table S2 lists the genes and coefficients used to calculate each subject’s risk score. Supplementary File S2 shows the corrected gene expression levels of TCGA and GSE13507 samples. The risk scores of BLCA patients in GSE13507 and TCGA were evaluated, and all patients were divided into a high-risk group and low-risk group according to the median risk score (Figure 5C). Compared with the low-risk group, there was no doubt that the number of deaths in high-risk group was significantly higher (Figure 5D). Further investigation revealed a negative correlation between the risk score and OS for BLCA patients (Figure 5E). Survival analysis of patients with BLCA from TCGA and GSE13507 was also plotted using R packages (“survival”, “survminer”). The results showed that the survival time was significantly shorter in the high-risk group than in the low-risk group (Figure 5F, *p* < 0.001). Meanwhile, the results of time-dependent ROC revealed that the projected AUC for 1, 3, and 5 years was 0.690, 0.686, and 0.677, respectively (Figure 5G). The calibration chart demonstrated that the predicted value of risk characteristics was essentially in accordance with its actual value (Figure 5H).

### 3.6. Combination Analysis of TMB and IRPM

**Figure 6:**
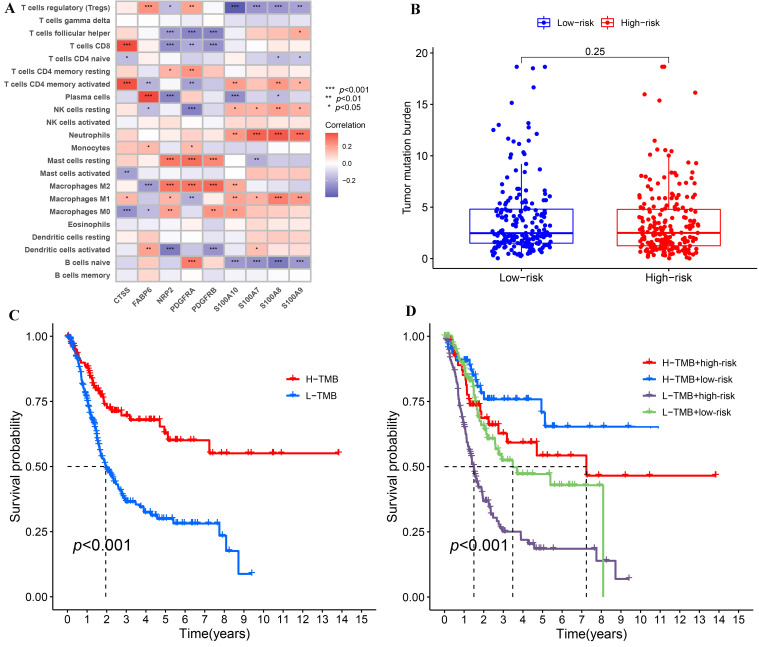
The correlations of IRPM-based risk characteristic with immune-cell infiltration and TMB. (**A**) Heatmap showed the correlations between 9 genes used to construct IRPM and 22 kinds of human immune cells. (**B**) Comparison of TMB between high-risk and low-risk groups in the TCGA-BLCA cohort. (**C**) Survival analysis based on the TMB in the TCGA-BLCA cohort. (**D**) Survival analysis for four groups stratified by combining the TMB and the IRPM-based risk characteristic in the TCGA-BLCA cohort.

We further analyzed IRPM using the data from TCGA-BLCA. Firstly, the Spearman’s correlations of nine genes used to calculate risk score with 22 kinds of human immune cells were analyzed by R packages (“limma”, “reshape2”, “tidyverse”). The results demonstrated that the nine genes were considerably correlated with many immune cells (Figure 6A), suggesting that the immune microenvironment played a momentous function in the prognosis of BLCA patients. Since TMB played an important role in the occurrence and development of many kinds of tumors [25,26], we explored the difference in TMB in high-risk and low-risk groups as well as the effect of TMB on the prognosis of BLCA. Although there was no substantial difference in TMB between the high-risk group and low-risk group (Figure 6B), the survival outcomes of high-TMB (H-TMB) BLCA patients were better than those of low-TMB (L-TMB) BLCA patients (Figure 6C), which indicated that TMB and risk score were not interfering with each other in influencing the prognosis of BLCA patients. We integrated TMB and risk score to further assess the prognosis of BLCA because both variables had a substantial impact on the patients’ prognosis. In comparison to other groups, the findings demonstrated that the survival outcome of the group (H-TMB + low-risk) was the best, whereas the survival outcome of the other group (L-TMB + high-risk) was the worst (Figure 6D).

### 3.7. Independent Prognostic Analysis of Risk Score and Clinicopathological Features and Construction and Validation of a Nomogram

**Figure 7:**
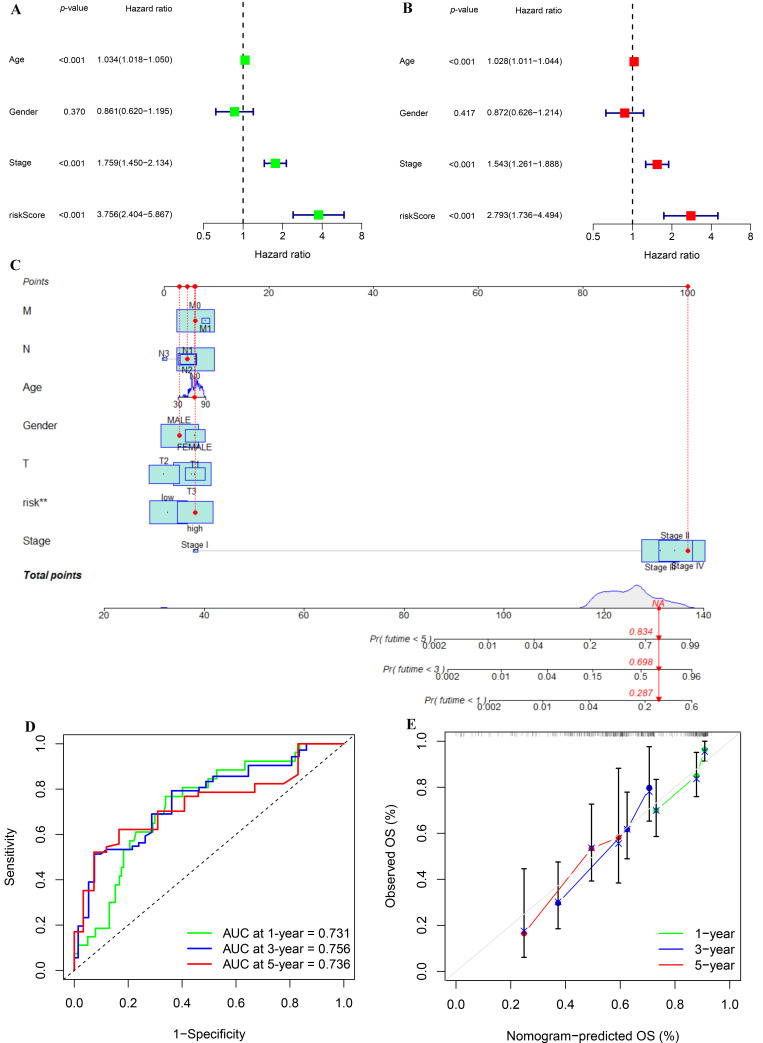
Univariate and multivariate Cox regression analyses and construction and analysis of a nomogram. (**A**) Forest plot of univariate Cox regression analysis in the TCGA-BLCA cohort. (**B**) Forest plot of multivariate Cox regression analysis in the TCGA-BLCA cohort. (**C**) Construction of a nomogram by combining IGPRM-based risk characteristic and clinicopathological information. (**D**) Prediction of 1-, 3-, and 5-year ROC and AUC for the nomogram in the TCGA-BLCA cohort. (**E**) The calibration diagram of the nomogram for TCGA-BLCA cohort.

As the risk score was significantly related to the development of BLCA, we explored whether the risk score could be utilized as an independent prognostic factor for BLCA by Cox regression analysis. The results showed that the risk score’s *p*-value was less than 0.001, and that its HR value was greater than the HR value of age and stage (Figure 7A,B), which demonstrated that risk score could not only be used as an independent prognostic predictor of BLCA, but also had a greater effect on the prognosis of BLCA than age and stage of patients. We also created a nomogram to make it easier for clinicians to assess the prognosis of BLCA (Figure 7C). Through the comprehensive assessment of clinicopathological feature and risk score, within a certain range, the higher the comprehensive score of patients, the worse the prognosis of patients. What is more, the ROC showed that the nomogram could effectively predict the BLCA patients’ survival outcome, and the AUC of 1 year, 3 years, and 5 years was 0.731, 0.756, and 0.736, respectively (Figure 7D). Last but not least, the calibration diagram demonstrated that the results obtained from the nomogram were quite reliable (Figure 7E).

### 3.8. Verification of the Effect of IRPM-Constructing Genes on the Prognosis of BLCA

**Figure 8:**
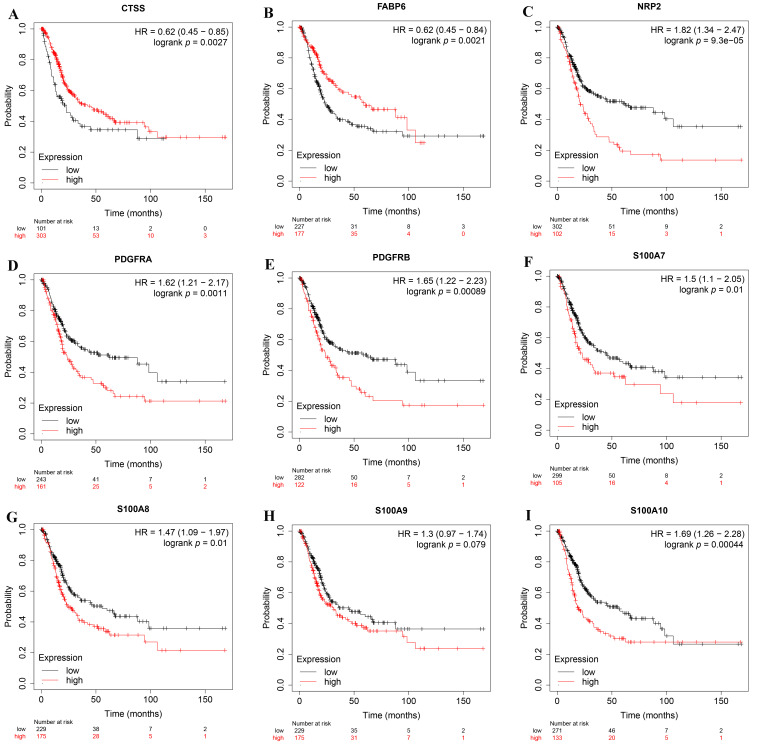
The expression levels of genes used to construct IRPM on the prognosis of BLCA using the Kaplan–Meier Plotter database. (**A**) CTSS; (**B**) FABP6; (**C**) NRP2; (**D**) PDGFRA; (**E**) PDGFRB; (**F**) S100A7; (**G**) S100A8; (**H**) S100A9; (**I**) S100A10.

In order to further deepen the credibility of our results, we verified the influence of IRPM-constructing genes on the prognosis of BLCA through the Kaplan–Meier Plotter database. The results showed that CTSS and FABP6 were low-risk genes (Figure 8A,B), while NRP2, PDGFRA, PDGFRB, S100A7, S100A8, S100A9, and S100A10 were high-risk genes (Figure 8C–I), and all of these genes significantly impacted the prognosis of BLCA except S100A9. These results were basically consistent with our results. Moreover, it is worth noting that we did not judge the prognosis of patients by single-gene expression, but by combining the expression of multiple genes, which undoubtedly made our results more reliable, and showed that the IRPM constructed by us may become a new method to assess the prognosis of BLCA.

### 3.9. Differences in the Expression of IRPM-Constructing Genes in BLCA Tissues and Adjacent Normal Tissues

**Figure 9:**
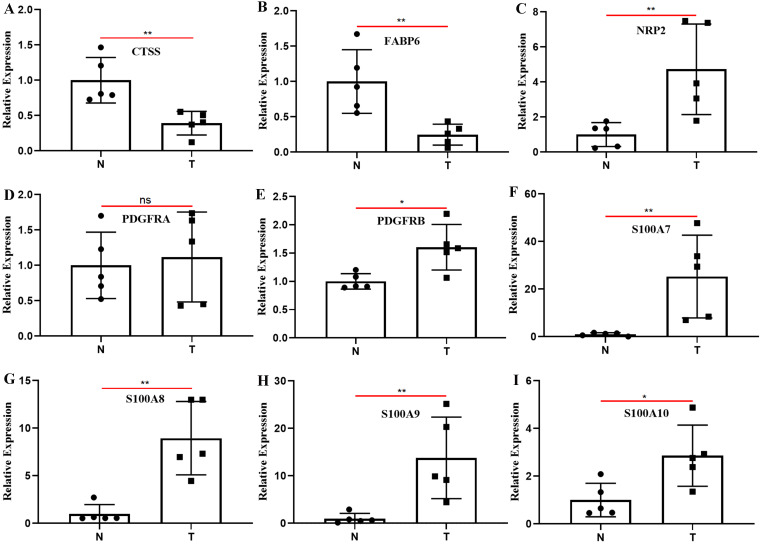
Clinical samples were used to detect the differences in the expression of nine genes used to construct IRPM between BLCA and adjacent normal tissues through RT-qPCR. (**A**) CTSS; (**B**) FABP6; (**C**) NRP2; (**D**) PDGFRA; (**E**) PDGFRB; (**F**) S100A7; (**G**) S100A8; (**H**) S100A9; (**I**) S100A10. T: BLCA tissues; N: adjacent normal tissues; * *p* < 0.05, ** *p* < 0.01; ns: the difference was not statistically significant.

We compared the expression levels of nine genes associated with constructing IRPM in BLCA and normal tissue adjacent to cancer using clinical samples. The RT-qPCR results revealed that almost all the IRPM-constructing genes were differentially expressed between BLCA and normal tissues adjacent to cancer, except PDGFRA (Figure 9A–I). The expressions of CTSS and FABP6 in BLCA tissues were significantly lower than those in adjacent normal tissues, and the expressions of NRP2, PDGFRB, S100A7, S100A8, S100A9, and S100A10 in BLCA tissues were significantly higher than those in adjacent normal tissues.

## 4. Discussion

BLCA is one of the major malignant tumors that seriously affects human health and quality of life [27]. In order to better treat BLCA and judge the prognosis of BLCA, we need to constantly explore and improve the molecular typing scheme of BLCA. Understanding the heterogeneity of TME is important for tumor prognosis and the effectiveness of targeted therapy [28,29,30], and the immune characteristics of tumors are an important part of TME. By mining the data from TCGA and GEO, this study aimed to realize the immune features of BLCA, to promote the study of molecular typing and prognosis of BLCA, and to help clinicians to more conveniently and precisely estimate the prognosis of BLCA, as well as to generate individualized treatment plans for BLCA patients.

Firstly, we identified two subtypes of BLCA, Immunity_H and Immunity_L, and examined their molecular features, including immune-cell infiltration, DEGs, and pathway enrichment. In comparison to Immunity_L, there was lower TumorPurity, higher fractions of immune cells, higher HLA gene expression, and richer immune-related pathways in Immunity_H, suggesting that the immunogenicity of Immunity_H was stronger, and revealing a potential relationship between immunogenicity and TumorPurity in BLCA. Studies have shown that HLA genes exert a momentous function in the recognition of cancer cells and in antitumor activity [31,32]. Activation of immune cells also plays an important role in the elimination of tumor cells [33,34]. Immunotherapy is likely to become the key method in the clinical treatment of many kinds of tumors [35]. These results better confirm the accuracy of our results. Moreover, it also means that the classification of BLCA into two subtypes is of great significance to distinguish the immunogenicity of BLCA.

On the other hand, the survival data and gene expression data of the TCGA-BLCA and GSE13507 were combined to identify 13 PIGs that may significantly affect the prognosis of BLCA patients, namely, CTSS, PDGFRA, PDGFRB, PDGFC, TNC, EDNRA, NRP2, TGFB3, FABP6, S100A7, S100A8, S100A9, and S100A10. Co-expression analysis and interaction network analysis of PIGs and DETFs were performed, which laid the foundation for studying the mechanism of these PIGs affecting the prognosis of BLCA. We constructed IRPM through the expression levels of nine genes in 13 PIGs. The nine genes were CTSS, PDGFRA, PDGFRB, FABP6, NRP2, S100A7, S100A8, S100A9, and S100A10. Previous studies have reported the role of the genes we used to construct IRPM in a variety of tumors. For instance, CTSS is a kind of lysosomal protease, the abnormal expression and activity of CTSS are related to the pathogenesis of many diseases, including cancer, cardiovascular disease, and diabetes [36], and CTSS may become a therapeutic target for lymphoma by altering the immune microenvironment of the tumor [37,38,39]. PDGFRA and PDGFRB exert a central function in the initiation and progression of many tumors, including glioblastoma, gastrointestinal stromal tumor, skin squamous cell carcinoma, and oral squamous cell carcinoma [40,41,42,43]. FABP6 has been reported as a potential target for inhibiting the progression of BLCA, and the mechanism may involve several aspects, such as affecting the activation of the AKT-mTOR signal and cell cycle [44]. NRP2 exerts a key role in lymphangiogenesis and lymphovascular invasion and affects the function of epithelial–mesenchymal transformation in the evolution of tumor transmission and metastasis [45,46]. Members of the S100 calcium binding protein family, namely, S100A7, S100A8, S100A9, and S100A10, exert a momentous function in tumor growth and metastasis, such as breast and liver cancer [47,48,49,50]. All these previous results prove that the nine genes used to calculate the risk score exert a momentous function in estimating the progression of cancer, and we jointly judge the development of BLCA based on these nine genes, which is undoubtedly more meaningful and accurate than judging the progression and prognosis of BLCA from the expression of a single gene. What is more, we verified the effect of the expressions of these nine genes on the prognosis of BLCA through the Kaplan–Meier Plotter database, and these results were basically consistent with our results. Last but not least, the results of RT-qPCR revealed that the expressions of CTSS and FABP6 in BLCA tissues were significantly lower than those in adjacent normal tissues, and the expressions of NRP2, PDGFRB, S100A7, S100A8, S100A9, and S100A10 in BLCA tissues were significantly higher than those in adjacent normal tissues. The results of RT-qPCR were similar to our previous prediction that CTSS and FABP6 were low-risk genes of BLCA, while NRP2, PDGFRA, PDGFRB, S100A7, S100A8, S100A9, and S100A10 were high-risk genes. The results of the Kaplan–Meier Plotter database and RT-qPCR demonstrated that the risk score has the potential to become a biomarker for judging BLCA progression and prognosis, and the genes used to calculate the risk score may become targets for the treatment of BLCA.

Since TMB has a crucial impact on the prognosis of a variety of tumors and is closely related to immunotherapy [51,52,53], we analyzed the TMB of high-risk and low-risk groups, and there was no significant difference in TMB between the two groups, but our results showed that TMB had an impact on the prognosis of BLCA, so we comprehensively analyzed the impact of TMB and risk score on the prognosis of BLCA. This provided a more precise means for us to judge the prognosis of BLCA.

In the end, we demonstrated that the risk score could be employed as a standalone indicator judging the prognosis of BLCA, and the HR value of risk score was much higher than HR value of age and tumor stage, which indicated that it was more meaningful to evaluate the BLCA patients’ prognosis by risk score than by age and tumor stage. Similar results have been reported in other cancer studies [54,55]. A nomogram is recognized as a convenient method to speculate the survival probability of patients [56,57,58]. To facilitate clinicians to assess the prognosis of BLCA, we constructed a nomogram based on comprehensive assessment of clinicopathological data and risk score, and validated its dependability by ROC and calibration chart, which demonstrated that the nomogram constructed by us was practical to some extent.

There is no doubt that our study has some shortcomings. First of all, all our data come from TCGA and GEO databases. In addition, our model lacks the verification of the cohort, but it should be noted that because we originally constructed the IRPM through the corrected data of GEO and TCGA, it is unreasonable to select a cohort for verification from the GEO, which requires us to verify the IRPM by observing the clinical cohort in the follow-up study. At present, there are many studies on constructing prognostic models from TCGA database and then verifying them through the cohort of GEO database [13,14,59], which is flawed. Since most of the cancer data are different in the GEO database and TCGA database, only the cancer prognosis model constructed by integrating the data of the two databases is more representative. The reason why we only compared the expression levels of these nine genes used to construct IRPM in five samples is to preliminarily verify the reliability of the model. Only by following up a large number of BLCA patients in the future and observing the correlation between the expression levels of these nine genes and the survival time of patients can we more accurately judge the reliability of the model constructed by us.

## 5. Conclusions

We divided BLCA into Immunity_H and Immunity_L subtypes, and explored the immune characteristics and molecular functions of the two subtypes in detail. In addition, we identified 13 immune genes related to the prognosis of BLCA, and constructed IRPM by using a novel data combination method through the expression of nine of these genes. The risk score has the potential to become a biomarker for judging BLCA progression and prognosis, and the genes used to calculate the risk score may become targets for the treatment of BLCA. In the meantime, we also constructed a nomogram to help clinicians more convenient to judge the survival outcome of BLCA. These results provided a reference for the treatment of BLCA and judging the prognosis of BLCA. Future studies need to verify the accuracy of IRPM constructed by us in predicting the prognosis of BLCA and its clinical application in the individualized treatment of BLCA.

## Figures and Tables

**Table 1:** The clinical information of the patients for RT-qPCR.

Sample	Sex	Years of Age	TNM Stage	Histological Type
Sample 1	Male	56	T_2a_N_0_M_0_	bladder urothelial cancer
Sample 2	Male	64	T_2b_N_0_M_0_	bladder urothelial cancer
Sample 3	Male	76	T_2a_N_0_M_0_	bladder urothelial cancer
Sample 4	Male	59	T_2a_N_0_M_0_	bladder urothelial cancer
Sample 5	Male	62	T_2b_N_0_M_0_	bladder urothelial cancer

## Data Availability

The datasets analyzed for this study can be found in here: TCGA (https://gdc.cancer.gov/), GEO (https://www.ncbi.nlm.nih.gov/geo/, GSE13507), ImmPort (https://www.immport.org/), and Kaplan–Meier Plotter database (https://kmplot.com/analysis/). The raw data of RT-qPCR will be made available by the authors, without undue reservation, to any qualified researcher.

## Supplementary Materials

The following supporting information can be downloaded at: https://www.mdpi.com/article/10.3390/biom12111670/s1. Table S1: The co-expression relationships between PIGs and DETFs. Table S2: The genes and coefficients used to calculate the risk score for each sample. Supplementary File S1: The signature genes representing 29 immune cells or immune-related functions. Supplementary File S2: The corrected gene expression level, survival time, and survival status of TCGA-BLCA and GSE13507 samples.

## References

[1] Siegel R.L., Miller K.D., Jemal A. (2020). Cancer statistics, 2020. CA Cancer J. Clin..

[2] Zhang M., Zhang X., Yu M., Zhang W., Zhang D., Zeng S., Wang X., Hu X. (2021). A Novel Ferroptosis-Related Gene Model for Overall Survival Predictions of Bladder Urothelial Carcinoma Patients. Front. Oncol..

[3] Warrick J.I., Sjodahl G., Kaag M., Raman J.D., Merrill S., Shuman L., Chen G., Walter V., DeGraff D.J. (2019). Intratumoral Heterogeneity of Bladder Cancer by Molecular Subtypes and Histologic Variants. Eur. Urol..

[4] Hoadley K.A., Yau C., Wolf D.M., Cherniack A.D., Tamborero D., Ng S., Leiserson M.D.M., Niu B., McLellan M.D., Uzunangelov V. (2014). Multiplatform analysis of 12 cancer types reveals molecular classification within and across tissues of origin. Cell.

[5] Witjes J.A., Bruins H.M., Cathomas R., Comperat E.M., Cowan N.C., Gakis G., Hernandez V., Linares Espinos E., Lorch A., Neuzillet Y. (2021). European Association of Urology Guidelines on Muscle-invasive and Metastatic Bladder Cancer: Summary of the 2020 Guidelines. Eur. Urol..

[6] Pfannstiel C., Strissel P.L., Chiappinelli K.B., Sikic D., Wach S., Wirtz R.M., Wullweber A., Taubert H., Breyer J., Otto W. (2019). The Tumor Immune Microenvironment Drives a Prognostic Relevance That Correlates with Bladder Cancer Subtypes. Cancer Immunol. Res..

[7] Hu J., Yu A., Othmane B., Qiu D., Li H., Li C., Liu P., Ren W., Chen M., Gong G. (2021). Siglec15 shapes a non-inflamed tumor microenvironment and predicts the molecular subtype in bladder cancer. Theranostics.

[8] Hu J., Othmane B., Yu A., Li H., Cai Z., Chen X., Ren W., Chen J., Zu X. (2021). 5mC regulator-mediated molecular subtypes depict the hallmarks of the tumor microenvironment and guide precision medicine in bladder cancer. BMC Med..

[9] Xu C., Pei D., Liu Y., Yu Y., Guo J., Liu N., Kang Z. (2022). Identification of a Novel Tumor Microenvironment Prognostic Signature for Bladder Urothelial Carcinoma. Front. Oncol..

[10] Da Costa J.B., Gibb E.A., Nykopp T.K., Mannas M., Wyatt A.W., Black P.C. (2018). Molecular tumor heterogeneity in muscle invasive bladder cancer: Biomarkers, subtypes, and implications for therapy. Urol. Oncol..

[11] Burrell R.A., McGranahan N., Bartek J., Swanton C. (2013). The causes and consequences of genetic heterogeneity in cancer evolution. Nature.

[12] Prasetyanti P.R., Medema J.P. (2017). Intra-tumor heterogeneity from a cancer stem cell perspective. Mol. Cancer.

[13] Kang Z., Li W., Yu Y.H., Che M., Yang M.L., Len J.J., Wu Y.R., Yang J.F. (2021). Identification of Immune-Related Genes Associated With Bladder Cancer Based on Immunological Characteristics and Their Correlation With the Prognosis. Front. Genet..

[14] Guan X., Xu Z.Y., Chen R., Qin J.J., Cheng X.D. (2020). Identification of an Immune Gene-Associated Prognostic Signature and Its Association With a Poor Prognosis in Gastric Cancer Patients. Front. Oncol..

[15] Cao R., Yuan L., Ma B., Wang G., Tian Y. (2020). Immune-related long non-coding RNA signature identified prognosis and immunotherapeutic efficiency in bladder cancer (BLCA). Cancer Cell Int..

[16] Wang Z., Tu L., Chen M., Tong S. (2021). Identification of a tumor microenvironment-related seven-gene signature for predicting prognosis in bladder cancer. BMC Cancer.

[17] Zhang L.H., Li L.Q., Zhan Y.H., Zhu Z.W., Zhang X.P. (2021). Identification of an IRGP Signature to Predict Prognosis and Immunotherapeutic Efficiency in Bladder Cancer. Front. Mol. Biosci..

[18] Hanzelmann S., Castelo R., Guinney J. (2013). GSVA: Gene set variation analysis for microarray and RNA-seq data. BMC Bioinform..

[19] Yoshihara K., Shahmoradgoli M., Martinez E., Vegesna R., Kim H., Torres-Garcia W., Trevino V., Shen H., Laird P.W., Levine D.A. (2013). Inferring tumour purity and stromal and immune cell admixture from expression data. Nat. Commun..

[20] Newman A.M., Liu C.L., Green M.R., Gentles A.J., Feng W., Xu Y., Hoang C.D., Diehn M., Alizadeh A.A. (2015). Robust enumeration of cell subsets from tissue expression profiles. Nat. Methods.

[21] Bhattacharya S., Dunn P., Thomas C.G., Smith B., Schaefer H., Chen J., Hu Z., Zalocusky K.A., Shankar R.D., Shen-Orr S.S. (2018). ImmPort, toward repurposing of open access immunological assay data for translational and clinical research. Sci. Data.

[22] Ritchie M.E., Phipson B., Wu D., Hu Y., Law C.W., Shi W., Smyth G.K. (2015). limma powers differential expression analyses for RNA-sequencing and microarray studies. Nucleic Acids Res..

[23] Zheng R., Wan C., Mei S., Qin Q., Wu Q., Sun H., Chen C.H., Brown M., Zhang X., Meyer C.A. (2019). Cistrome Data Browser: Expanded datasets and new tools for gene regulatory analysis. Nucleic Acids Res..

[24] Mei S., Qin Q., Wu Q., Sun H., Zheng R., Zang C., Zhu M., Wu J., Shi X., Taing L. (2017). Cistrome Data Browser: A data portal for ChIP-Seq and chromatin accessibility data in human and mouse. Nucleic Acids Res..

[25] Zhang K., Qian Y., Quan X., Zhu T., Qian B. (2022). A Novel Signature of Lipid Metabolism-Related Gene Predicts Prognosis and Response to Immunotherapy in Lung Adenocarcinoma. Front. Cell Dev. Biol..

[26] Zou X., Guo B., Ling Q., Mo Z. (2022). Toll-Like Receptors Serve as Biomarkers for Early Diagnosis and Prognosis Assessment of Kidney Renal Clear Cell Carcinoma by Influencing the Immune Microenvironment: Comprehensive Bioinformatics Analysis Combined With Experimental Validation. Front. Mol. Biosci..

[27] Tang C., Ma J., Liu X., Liu Z. (2020). Development and validation of a novel stem cell subtype for bladder cancer based on stem genomic profiling. Stem. Cell Res. Ther..

[28] Hanahan D., Weinberg R.A. (2011). Hallmarks of cancer: The next generation. Cell.

[29] Hanahan D., Coussens L.M. (2012). Accessories to the crime: Functions of cells recruited to the tumor microenvironment. Cancer Cell.

[30] Schulz M., Salamero-Boix A., Niesel K., Alekseeva T., Sevenich L. (2019). Microenvironmental Regulation of Tumor Progression and Therapeutic Response in Brain Metastasis. Front. Immunol..

[31] Sadagopan A., Michelakos T., Boyiadzis G., Ferrone C., Ferrone S. (2022). Human Leukocyte Antigen Class I Antigen-Processing Machinery Upregulation by Anticancer Therapies in the Era of Checkpoint Inhibitors: A Review. JAMA Oncol..

[32] Kawazu M., Ueno T., Saeki K., Sax N., Togashi Y., Kaneseki T., Chida K., Kishigami F., Sato K., Kojima S. (2021). HLA Class I Analysis Provides Insight Into the Genetic and Epigenetic Background of Immune Evasion in Colorectal Cancer With High Microsatellite Instability. Gastroenterology.

[33] Li K., Zhang Z., Mei Y., Li M., Yang Q., Wu Q., Yang H., He L., Liu S. (2022). Targeting the innate immune system with nanoparticles for cancer immunotherapy. J. Mater. Chem. B.

[34] Chaturvedi P., George V., Shrestha N., Wang M., Dee M.J., Zhu X., Liu B., Egan J., D’Eramo F., Spanoudis C. (2022). Immunotherapeutic HCW9218 augments anti-tumor activity of chemotherapy via NK cell-mediated reduction of therapy-induced senescent cells. Mol. Ther..

[35] Chen D.S., Mellman I. (2013). Oncology meets immunology: The cancer-immunity cycle. Immunity.

[36] Brown R., Nath S., Lora A., Samaha G., Elgamal Z., Kaiser R., Taggart C., Weldon S., Geraghty P., Cathepsin S. (2020). Investigating an old player in lung disease pathogenesis, comorbidities, and potential therapeutics. Respir. Res..

[37] Riether C., Ochsenbein A.F. (2020). Genetic Alterations Impact Immune Microenvironment Interactions in Follicular Lymphoma. Cancer Cell.

[38] Dheilly E., Battistello E., Katanayeva N., Sungalee S., Michaux J., Duns G., Wehrle S., Sordet-Dessimoz J., Mina M., Racle J. (2020). Cathepsin S Regulates Antigen Processing and T Cell Activity in Non-Hodgkin Lymphoma. Cancer Cell.

[39] Bararia D., Hildebrand J.A., Stolz S., Haebe S., Alig S., Trevisani C.P., Osorio-Barrios F., Bartoschek M.D., Mentz M., Pastore A. (2020). Cathepsin S Alterations Induce a Tumor-Promoting Immune Microenvironment in Follicular Lymphoma. Cell Rep..

[40] Aldaz P., Arozarena I. (2021). Tyrosine Kinase Inhibitors in Adult Glioblastoma: An (Un)Closed Chapter?. Cancers.

[41] Bernat-Peguera A., Simón-Extremera P., da Silva-Diz V., López de Munain M., Díaz-Gil L., Penin R.M., González-Suárez E., Pérez Sidelnikova D., Bermejo O., Viñals J.M. (2019). PDGFR-induced autocrine SDF-1 signaling in cancer cells promotes metastasis in advanced skin carcinoma. Oncogene.

[42] Hayashi Y., Bardsley M.R., Toyomasu Y., Milosavljevic S., Gajdos G.B., Choi K.M., Reid-Lombardo K.M., Kendrick M.L., Bingener-Casey J., Tang C.M. (2015). Platelet-Derived Growth Factor Receptor-α Regulates Proliferation of Gastrointestinal Stromal Tumor Cells With Mutations in KIT by Stabilizing ETV1. Gastroenterology.

[43] Lin L.H., Lin J.S., Yang C.C., Cheng H.W., Chang K.W., Liu C.J. (2020). Overexpression of Platelet-Derived Growth Factor and Its Receptor Are Correlated with Oral Tumorigenesis and Poor Prognosis in Oral Squamous Cell Carcinoma. Int. J. Mol. Sci..

[44] Lin C.H., Chang H.H., Lai C.R., Wang H.H., Tsai W.C., Tsai Y.L., Changchien C.Y., Cheng Y.C., Wu S.T., Chen Y. (2022). Fatty Acid Binding Protein 6 Inhibition Decreases Cell Cycle Progression, Migration and Autophagy in Bladder Cancers. Int. J. Mol. Sci..

[45] Poghosyan S., Frenkel N., Lentzas A., Laoukili J., Rinkes I.B., Kranenburg O., Hagendoorn J. (2022). Loss of Neuropilin-2 in Murine Mesenchymal-like Colon Cancer Organoids Causes Mesenchymal-to-Epithelial Transition and an Acquired Dependency on Insulin-Receptor Signaling and Autophagy. Cancers.

[46] Lungulescu C., Ghimpau V., Gheonea D.I., Sur D., Lungulescu C.V. (2022). The Role of Neuropilin-2 in the Epithelial to Mesenchymal Transition of Colorectal Cancer: A Systematic Review. Biomedicines.

[47] Mishra S., Charan M., Shukla R.K., Agarwal P., Misri S., Verma A.K., Ahirwar D.K., Siddiqui J., Kaul K., Sahu N. (2022). cPLA2 blockade attenuates S100A7-mediated breast tumorigenicity by inhibiting the immunosuppressive tumor microenvironment. J. Exp. Clin. Cancer Res..

[48] Lu Z., Zheng S., Liu C., Wang X., Zhang G., Wang F., Wang S., Huang J., Mao S., Lei Y. (2021). S100A7 as a potential diagnostic and prognostic biomarker of esophageal squamous cell carcinoma promotes M2 macrophage infiltration and angiogenesis. Clin. Transl. Med..

[49] Goh J.Y., Feng M., Wang W., Oguz G., Yatim S., Lee P.L., Bao Y., Lim T.H., Wang P., Tam W.L. (2017). Chromosome 1q21.3 amplification is a trackable biomarker and actionable target for breast cancer recurrence. Nat. Med..

[50] Zhou X., Shi M., Cao J., Yuan T., Yu G., Chen Y., Fang W., Li H. (2021). S100 Calcium Binding Protein A10, A Novel Oncogene, Promotes the Proliferation, Invasion, and Migration of Hepatocellular Carcinoma. Front. Genet..

[51] Chan T.A., Yarchoan M., Jaffee E., Swanton C., Quezada S.A., Stenzinger A., Peters S. (2019). Development of tumor mutation burden as an immunotherapy biomarker: Utility for the oncology clinic. Ann. Oncol..

[52] Palmeri M., Mehnert J., Silk A.W., Jabbour S.K., Ganesan S., Popli P., Riedlinger G., Stephenson R., de Meritens A.B., Leiser A. (2021). Real-world application of tumor mutational burden-high (TMB-high) and microsatellite instability (MSI) confirms their utility as immunotherapy biomarkers. ESMO Open.

[53] Jiang T., Chen J., Xu X., Cheng Y., Chen G., Pan Y., Fang Y., Wang Q., Huang Y., Yao W. (2022). On-treatment blood TMB as predictors for camrelizumab plus chemotherapy in advanced lung squamous cell carcinoma: Biomarker analysis of a phase III trial. Mol. Cancer.

[54] Zhao Y., Song Q., Xu F., Zhou Y., Zuo X., Zhang Z. (2022). Pyroptosis-Related Risk Signature Exhibits Distinct Prognostic, Immune, and Therapeutic Landscapes in Hepatocellular Carcinoma. Front. Genet..

[55] Li H., Zhang X., Yi C., He Y., Chen X., Zhao W., Yu D. (2021). Ferroptosis-related gene signature predicts the prognosis in Oral squamous cell carcinoma patients. BMC Cancer.

[56] Fakhry C., Zhang Q., Nguyen-Tan P.F., Rosenthal D.I., Weber R.S., Lambert L., Trotti A.M., Barrett W.L., Thorstad W.L., Jones C.U. (2017). Development and Validation of Nomograms Predictive of Overall and Progression-Free Survival in Patients With Oropharyngeal Cancer. J. Clin. Oncol..

[57] Wang S.J., Fuller C.D., Kim J.S., Sittig D.F., Thomas C.R., Ravdin P.M. (2008). Prediction model for estimating the survival benefit of adjuvant radiotherapy for gallbladder cancer. J. Clin. Oncol..

[58] Previs R.A., Bevis K.S., Huh W., Tillmanns T., Perry L., Moore K., Chapman J., McClung C., Kiet T., Java J. (2014). A prognostic nomogram to predict overall survival in women with recurrent ovarian cancer treated with bevacizumab and chemotherapy. Gynecol. Oncol..

[59] Yuan K., Zeng R., Deng P., Zhang A., Liu H., Wang N., Tang Y., Yin Z., Liu H. (2022). Identification and Verification of Immune-Related Genes Prognostic Signature Based on ssGSEA for Adrenocortical Carcinoma (ACC). Int. J. Gen. Med..

