# Supplementary material for: Identification of Immune-Related Subtypes and Construction of a Novel Prognostic Model for Bladder Urothelial Cancer
Source: Biomolecules. 2022 Nov 11;12(11):1670. doi: 10.3390/biom12111670 (PMC9687876; doi:10.3390/biom12111670)
Supplement: Supplementary file 1 [file biomolecules-12-01670-s001.zip › Supplementray table S1.pdf]

**Table S1：**

The co-expression relationships between PIGs and DETFs

| PIGs | DETFs | cor | *p*-value | FDR |
|---|---|---|---|---|
| CTSS | IKZF1 | 0.454968 | <0.001 | <0.001 |
| CTSS | FOXP3 | 0.488266 | <0.001 | <0.001 |
| CTSS | IRF1 | 0.536781 | <0.001 | <0.001 |
| CTSS | CIITA | 0.493997 | <0.001 | <0.001 |
| CTSS | STAT1 | 0.544606 | <0.001 | <0.001 |
| CTSS | FLI1 | 0.418201 | <0.001 | <0.001 |
| PDGFRB | NFATC1 | 0.457551 | <0.001 | <0.001 |
| PDGFRB | ETS1 | 0.404492 | <0.001 | <0.001 |
| PDGFRB | GATA6 | 0.446763 | <0.001 | <0.001 |
| PDGFRB | ERG | 0.454894 | <0.001 | <0.001 |
| PDGFRB | FLI1 | 0.446423 | <0.001 | <0.001 |
| PDGFRB | WWTR1 | 0.528426 | <0.001 | <0.001 |
| PDGFRB | MITF | 0.452607 | <0.001 | <0.001 |
| PDGFC | MAF | 0.448408 | <0.001 | <0.001 |
| PDGFC | NR3C1 | 0.457348 | <0.001 | <0.001 |
| PDGFC | WWTR1 | 0.585299 | <0.001 | <0.001 |
| PDGFC | MITF | 0.426719 | <0.001 | <0.001 |
| TNC | SNAI2 | 0.460336 | <0.001 | <0.001 |
| TNC | TEAD4 | 0.411191 | <0.001 | <0.001 |
| EDNRA | ETS1 | 0.423822 | <0.001 | <0.001 |
| EDNRA | GATA6 | 0.479072 | <0.001 | <0.001 |
| EDNRA | ERG | 0.482777 | <0.001 | <0.001 |
| EDNRA | FLI1 | 0.414418 | <0.001 | <0.001 |
| EDNRA | WWTR1 | 0.587373 | <0.001 | <0.001 |
| EDNRA | MITF | 0.428804 | <0.001 | <0.001 |
| NRP2 | GATA6 | 0.513843 | <0.001 | <0.001 |
| NRP2 | ERG | 0.400806 | <0.001 | <0.001 |
| NRP2 | GATA3 | -0.40846 | <0.001 | <0.001 |
| NRP2 | WWTR1 | 0.603609 | <0.001 | <0.001 |
| NRP2 | FOXA1 | -0.40036 | <0.001 | <0.001 |
| NRP2 | MITF | 0.441295 | <0.001 | <0.001 |
| PDGFRA | SOX17 | 0.445718 | <0.001 | <0.001 |
| PDGFRA | FLI1 | 0.425145 | <0.001 | <0.001 |
| PDGFRA | WWTR1 | 0.497123 | <0.001 | <0.001 |
| PDGFRA | MEF2C | 0.470663 | <0.001 | <0.001 |
| TGFB3 | MYH11 | 0.426813 | <0.001 | <0.001 |
| TGFB3 | GATA6 | 0.579091 | <0.001 | <0.001 |
| TGFB3 | ERG | 0.432887 | <0.001 | <0.001 |
| TGFB3 | WWTR1 | 0.596307 | <0.001 | <0.001 |
| TGFB3 | MITF | 0.495075 | <0.001 | <0.001 |
| S100A10 | CEBPB | 0.411628 | <0.001 | <0.001 |

| | | | | |
|---|---|---|---|---|
| S100A10 | TEAD4 | 0.410409 | <0.001 | <0.001 |
| S100A10 | GATA3 | -0.40032 | <0.001 | <0.001 |
| S100A10 | FOXA1 | -0.47654 | <0.001 | <0.001 |
