# Supplementary material for: Identification of Immune-Related Subtypes and Construction of a Novel Prognostic Model for Bladder Urothelial Cancer
Source: Biomolecules. 2022 Nov 11;12(11):1670. doi: 10.3390/biom12111670 (PMC9687876; doi:10.3390/biom12111670)
Supplement: Supplementary file 1 [file biomolecules-12-01670-s001.zip › Supplementray table S2.pdf]

Table S2:

The genes and coefficients used to calculate the risk score for each sample

Gene	Coefficient
CTSS	-0.207238438969665
S100A7	0.0135458074306028
PDGFRB	0.0637450037740906
NRP2	0.099640691165939
PDGFRA	0.116554628802348
S100A10	0.000282049731445765
S100A9	0.00828914769197644
FABP6	-0.047309990518725
S100A8	0.0386435659526242
